# Guideline Approaches for Cardioendocrine Disease Surveillance and Treatment Following Spinal Cord Injury

**DOI:** 10.1007/s40141-018-0203-z

**Published:** 2018-11-15

**Authors:** Mark S. Nash, James L. J. Bilzon

**Affiliations:** 1 The Miami Project to Cure Paralysis, University of Miami Miller School of Medicine, Miami, FL USA; 2 Departments of Neurological Surgery and Physical Medicine & Rehabilitation, University of Miami Miller School of Medicine, Miami, FL USA; 3 Department for Health, University of Bath, Bath, Somerset UK

**Keywords:** Cardioendocrine disease, Spinal cord injury, Nutrition, Exercise, Physical activity, Pharmacotherapy, Bariatrics

## Abstract

**Purpose of Review:**

Persons with spinal cord injuries (SCI) commonly experience individual risks and coalesced health hazards of the cardiometabolic syndrome (CMS). This review will examinethe role of exercise and nutritional intervention as countermeasures to these disease risks.

**Recent Findings:**

The CMS hazards of overweight/obesity, insulin resistance, hypertension, and dyslipidemia are strongly associated with physical deconditioning and are common after SCI. Both the CMS diagnosis and physical deconditioning worsen the prognosis for all-cause cardiovascular disease occurring early after SCI. Evidence supports a therapeutic role for physical activity after SCI as an effective countermeasure to these risks and often represents the first-line approach to CMS abatement. This evidence is supported by authoritative systematic reviews and associated guidelines that recommend specific activities, frequencies, and activities of work. In many cases, the most effective exercise programming uses more intense periods of work with limited rest. As SCI is also associated with poor dietary habits, including excessive energy intake and saturated fat consumption, more comprehensive lifestyle management incorporating both exercise and nutrition represents a preferred approach for overall health management.

**Summary:**

Irrespective of the interventional strategy, improved surveillance of the population for CMS risks and encouraged incorporation of exercise and nutritional management according to recent population-specific guidelines will most likely play an important role in the preservation of activity, optimal health, and independence throughout the lifespan.

## Cardioendocrine Disease

### The Cardiometabolic Syndrome (CMS)

**Fig. 1:**
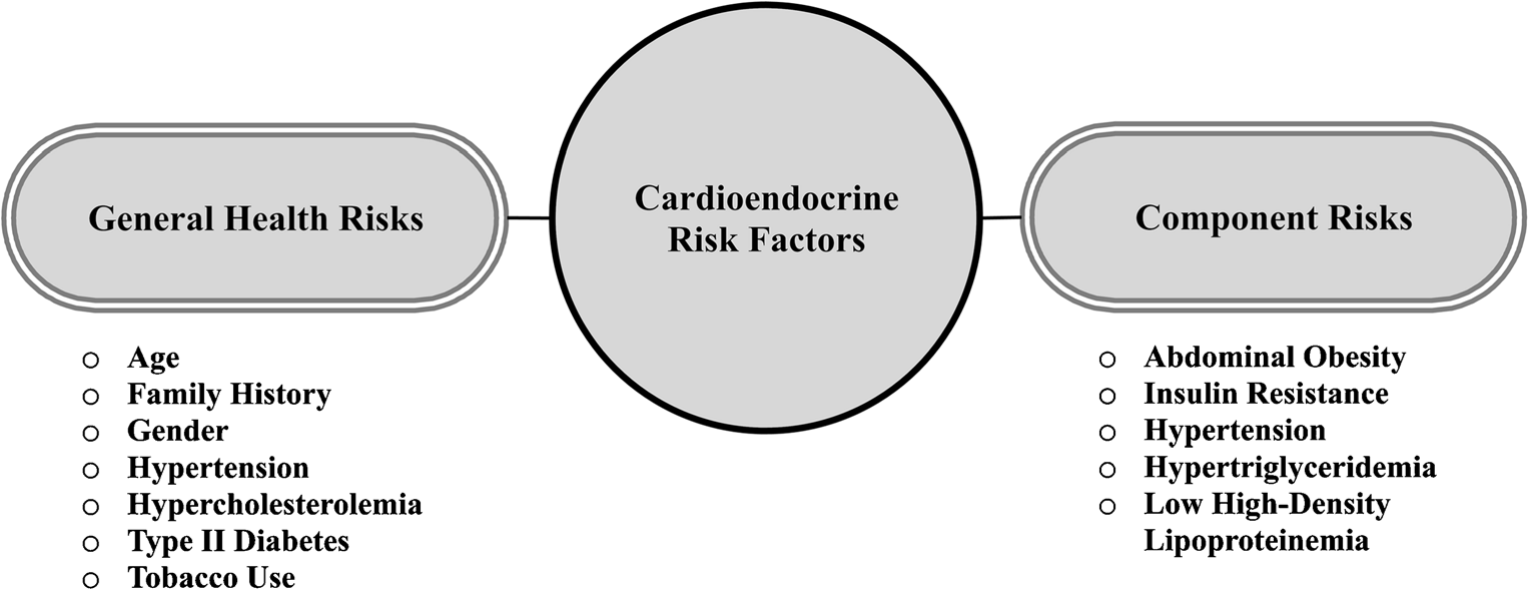
Cardioendocrine health risks

The CMS—also known as “syndrome X,” insulin resistance syndrome, Reaven’s syndrome, and metabolic syndrome—is a coalescing of cardiovascular, renal, metabolic, pro-thrombotic, and inflammatory health risks [1]. Figure 1 shows the general health indicators and component risks for the CMS. Co-occurrence of three (or more) of the following health risks typically defines the CMS: abdominal (central) obesity, hypertension, insulin resistance, and dyslipidemia, the latter as either hypertriglyceridemia or low high-density lipoproteinemia. When so co-expressed, these risks are recognized as a *distinct disease entity* by the American Society of Endocrinology, American Heart Association (AHA), International Diabetes Federation (IDF), NIH National Heart Lung Blood Institute (NIH-NHLBI), and the World Health Organization (WHO) [2]. While the definitions for CMS shown in Table 1 have yet to be harmonized entirely [3], it is consistently recognized that *any* coalescing of risk factors worsens a cardiovascular disease (CVD) prognosis. In particular, a CMS diagnosis increases the odds of developing atherosclerotic disease, heart failure, and diabetes and poses a health risk equivalent to that of either type 2 diabetes or existing coronary disease. CMS is currently reported in 22.9% of the U.S. adult population [4] and is increasing at a rate that resembles a pandemic of communicable diseases.

**Table 1:** Commonly used guideline definitions for the CMS

Authority	Diagnosis	
IDF (2006) [5, 6]	Central obesity (defined as waist circumference^#^ with ethnicity-specific values) AND any two of: ^#^note: central obesity is assumed if BMI > 30 kg/m^2^	TG triglycerides: > 150 mg/dL (1.7 mmol/L) or treatment for elevated TG
		HDL cholesterol: < 40 mg/dL (1.03 mmol/L) in males, < 50 mg/dL (1.29 mmol/L) in females, or treatment for low HDL
		Raised blood pressure (BP): systolic BP > 130 or diastolic BP > 85 mmHg, or treatment of previously diagnosed hypertension
		Raised fasting plasma glucose (FPG): > 100 mg/dL (5.6 mmol/L), or previously diagnosed type 2 diabetes
NCEP (2002) [7] and AHA/NHLBI (2004) [8, 9]	At least three of: NOTE: NCEP and AHA/NHLBI are identical except for the AHA definition of fasting plasma glucose	Waist circumference: • Men—greater than 40 in. (102 cm) • Women—greater than 35 in. (88 cm)
		Plasma triglycerides: ≥ 150 mg/dL (1.7 mmol/L)
		Reduced HDL (“good”) cholesterol: Men—less than 40 mg/dL (1.03 mmol/L) Women—Less than 50 mg/dL (1.29 mmol/L)
		Elevated blood pressure: equal to or greater than 130/85 mmHg or use of medication for hypertension
		Fasting glucose: ≥ 110 mg/dL (6.1 mmol/L) or use of medication for hyperglycemia AHA: Fasting glucose ≥ 100 mg/dL (5.6 mmol/L) or use of medication for hyperglycemia
WHO (1998) [10]	Any of diabetes mellitus, impaired glucose tolerance (IFG), impaired fasting glucose or insulin resistance, AND two of the following: NOTE: IFG is two-hour glucose levels of 140 to 199 mg per dL (7.8 to 11.0 mmol/l) on the 75-g oral glucose tolerance test	Blood pressure ≥ 140/90 mmHg
		Triglycerides (TG) ≥ 1.695 mmol/L and high-density lipoprotein cholesterol (HDL-C) ≤ 0.9 mmol/L (male), ≤ 1.0 mmol/L (female)
		Central obesity: waist:hip ratio > 0.90 (male); > 0.85 (female), or body mass index > 30 kg/m^2^
		Microalbuminuria: urinary albumin excretion ratio ≥ 20 μg/min or albumin:creatinine ratio ≥ 30 mg/g

### Prevalence and Causes of CMS After SCI

CMS develops from a mismatch between daily energy intake and energy expenditure [5], making persons with SCI a high-risk target for the disorder. The principal *metabolic abnormality* of the syndrome is insulin resistance, while the unified *cause* ensues excessive body adipose mass associated with visceral and ectopic fat depots. Combined “overweight” and “obesity” in persons with chronic SCI describes 60–80% of the population [6, 7], with the most common period for gain in body mass occurring at 2–7 months after completion of post-injury rehabilitation [8, 9]. Not surprisingly, the component risks of CMS are not equally weighted, with sarcopenic obesity [10]—a highly prevalent finding after SCI [11–15]—appearing to be the most powerful progenitor, followed by insulin resistance.

Beyond the characteristic findings of sarcopenic obesity and insulin resistance, all-cause disorders of the integrated cardioendocrine system have been reported in persons with SCI since the early 1980s [16–18] and are thought to hasten cardiovascular-related morbidity and mortality [15, 19–21]. The genesis of these disorders is primarily attributed to CMS risk factors observed in the non-disabled population, although reported at a significantly elevated prevalence after SCI [6]. These risks include widely cited atherogenic dyslipidemia with low levels of the cardioprotective high-density lipoprotein cholesterol (HDL-C), [22–27] dyslipidemia attributed to immobilization-related physical deconditioning [28], and frequently associated with sarcopenia [29] and diminished resting energy expenditure [30, 31]. Otherwise, inadequate caloric expenditure by lowered daily resting energy expenditure and physical activity energy expenditure is thought to increase body fat mass, which is considered a sine qua non of CMS after SCI [32, 33].

### Non-Guideline CMS Risks of Sedentary Lifestyle and Imprudent Nutrition After SCI

While physical deconditioning per se is not included among the five component risks of CMS, it is linked with and considered a significant cause of, obesity, insulin resistance, hypertension, and dyslipidemia [15, 34]. The same can be assumed for a hypercaloric diet relative to daily need [8, 35]. Several factors point to physical deconditioning after SCI as a significant contributor to a CMS diagnosis. First, the SCI population was long ago identified at the lowest end of the human fitness continuum, making physical deconditioning suspect as a cause for CMS-related risks [23, 36–38]. Second, a common finding after SCI is a low HDL-C, [22, 24, 39] which is strongly linked with low levels of cardiorespiratory fitness in persons without disability [40–42]. Third, barriers to exercise participation are common after SCI and may include either self-imposed obstacles to exercise participation or those associated with legitimate physical barriers to exercise, lack of adapted exercise equipment, limited professional assistance, societal mores, and financial limitations [43–47].

In addition to physical inactivity, CMS in humans is strongly influenced by dietary habits and nutritional status [48]. The latter may be significantly altered after SCI due to changes in the metabolic milieu (e.g., loss of metabolically active tissue), physical barriers (e.g., access to food shopping and grocery store shelving), environment (e.g., institutional food), functional challenges (e.g., difficulties encountered in preparing food), and social factors (e.g., food provided as comfort by family/friends) [49]. As persons with SCI living in what has been termed an “obesogenic environment,” [49] this reality and other factors combine to make lifelong healthy nutrition habits all the more challenging.

With respect to nutritional intake, data reported since 2008 indicate that men with SCI consume 500–600 fewer kilocalories than the ∼ 2600 kcal standard for men in the general population [8, 50], while caloric intake for women with SCI is about the same or slightly (∼ 100 kcal) lower than the expected intake of ∼ 1800 kcal [51, 52]. However, data comparing resting energy expenditure and average daily caloric ingestion *still identify a surplus intake* of ∼ 300–500 kcal per day [52–54]. Although this excess intake may seem inconsequential, even a small, sustained caloric excess will eventually lead to weight gain, pathogenic lipid profiles, impaired glycemic control, disease, and increased mortality. More precise data on total caloric expenditure relative to total energy expenditure are thus needed to fashion specific dietary recommendations for persons with SCI and emphasize the need for better matching of caloric intake and expenditure as a primary goal of a healthy post-SCI lifestyle [55, 56].

Despite a lower total daily energy intake than the general population, many studies also report that persons with SCI consume relatively more dietary fat than is recommended [8, 35, 51, 52, 57, 58]. In particular, saturated fat intakes are at the high end of, or exceed the recommended limit (typically < 10% of total calories) [8, 35, 57, 58], although may decline with the passage of time [59]. High-fat intake is commonly associated with weight gain, and in particular, high dietary levels of saturated fat adversely affect metabolic profiles and chronic disease outcomes [60, 61]. There is also evidence for a direct relationship between high-fat intake and serum triglycerides (TGs) after SCI [59], as well as elevated body mass index [39]. The high-fat gain may also worsen a reported exaggerated postprandial lipemia in persons with SCI, [62, 63] in which remnant lipoproteins from delayed metabolism of dietary TGs may accelerate the transfer of TG-rich lipoproteins in the vascular wall and hasten atherogenesis [64].

### Guidelines for Addressing CMS Risks After SCI

Given the well-documented CMS risks after SCI and the lack of a unified treatment strategy for its composite and individualized risks, the Consortium for Spinal Cord Medicine recently convened an expert panel to develop *Guidelines for Identification and Management of Cardiometabolic Risk after Spinal Cord Injury* [65]. These guidelines (from now on the “PVA Guidelines”), and others will form the basis for the remaining information presented in this monograph.

## Definition and Surveillance of CMS

The PVA Guidelines [65] recommend the use of the AHA definition for determining CMS in persons with SCI (Table 2). As waist circumference is not a validated proxy for obesity in SCI, the PVA Guideline assumed definitions of obesity as: (a) > 22% body fat when using 3- or 4-compartment modeling or (b) BMI ≥ 22 kg/m^2^. Table 3 identifies timing for post-injury surveillance and periodic follow-up for the CMS diagnosis and component risks.

**Table 2:** PVA Guideline definition of the CMS

Authority	Diagnosis	
AHA/NHLBI [3, 12]	Three or more of:	> 22% body fat when using 3- or 4-compartment modeling, or BMI ≥ 22 kg/m^2^
		Plasma TG: ≥ 150 mg/dL (1.7 mmol/L)
		Reduced HDL (“good”) cholesterol: • Men—less than 40 mg/dL (1.03 mmol/L) • Women—less than 50 mg/dL (1.29 mmol/L)
		Elevated blood pressure: ≥ 130/85 mmHg or use of medication for hypertension
		Fasting glucose ≥ 100 mg/dL (5.6 mmol/L) or use of medication for hyperglycemia

**Table 3:** Guidelines for testing of CMD and its five component risks at discharge from rehabilitation and follow-up

Risk	Test	Patients	Initial	Follow-up
CMD	3+ risk components (see below)	All	At discharge from rehabilitation	Annually
CMD risk components				
Impaired fasting glucose, pre-diabetes, and diabetes	FPG, OGTT, or A1C	Asymptomatic individuals with SCI having one or more risk factors	FBG annually; other tests at a minimum of 3-year intervals if tests are normal	
Obesity	Multi-compartment modeling or BMI	Individuals having confirmed pre-diabetes, diabetes, or CMD		Annual testing and ongoing management
Dyslipidemia	Fasting lipid panel preferred, but at minimum HDL-C and TG	All	At discharge from rehabilitation	Annual testing, or when evidence of elevated risk is identified
Hypertension	Blood pressure			Measured at every routine visit (and at least annually). Elevated BP readings should be confirmed on a separate visit to diagnose hypertension.
Lifestyle risk factors				
Suboptimal nutrition	Maintenance of stable body-fat mass or whole-body mass throughout the lifespan	All	Medically supervised nutrition plan beginning in rehabilitation, or as soon as possible	Continuous throughout the lifespan
Physical deconditioning	Exercise testing if practical	All, insofar as feasible and practical	Recommendations for therapeutic or recreational exercise initiated by the time of rehabilitation discharge	Annual with continuous follow-up throughout the lifespan

## Lifestyle Intervention

While physical activity has established benefits as a countermeasure to excessive energy intake, some persons with SCI cannot effectively balance energy intake and expenditure with physical activity alone. Some are limited by their level of injury [66] and overuse injuries [67, 68] as well as other documented barriers to exercise [43, 45, 69, 70]. Based on the existing evidence and appreciating that energy expenditure from upper-body physical activity rarely compensates for excessive caloric intake, nutritional modification may represent a favored target for obesity management and CMS prevention in individuals with SCI. The panel does not recommend a single nutritional plan but notes success in weight loss using the Mediterranean diet in the Diabetes Prevention Program [71, 72], and the DASH Diet, which may be more useful for hypertension management [73, 74]. The Healthy Mediterranean-Style Pattern is also adapted from the Healthy U.S.-Style Pattern, modifying amounts recommended from some food groups to reflect eating patterns that have been associated with positive health outcomes in studies of Mediterranean-style diets.

To date, prospective evaluation of weight loss programs in the SCI population has been limited. Weight loss programs designed for the non-disabled population may not be appropriate for the specific health [20, 55, 56, 75] and nutritional needs [8, 35, 49] of the SCI population. A pilot study of a weight loss program consisting of education on nutrition, exercise, and behavioral modification in individuals with chronic SCI who were overweight or obese resulted in weight loss and improvements in dietary intake [76]. This study utilized the time-calorie displacement diet, which emphasizes large intakes of high bulk, low energy-density foods, such as fruits and vegetables, high-fiber grains, and cereals. It also emphasized a moderate intake of high energy-density foods, such as meats, cheeses, sugars, and fats (Table 4).

**Table 4:** Risk targets for management of CMS through primary lifestyle intervention using nutrition and exercise

CMD risk	Goal	Primary management: lifestyle intervention	
		Nutrition	Exercise
CMs diagnosis	Reduce the number of risk components to < 3	Institute the following nutritional adjustments beginning as soon as possible after the SCI: 1. For all individuals, adopt a heart-healthy nutrition plan focusing on fruits, vegetables, whole grains, low-fat dairy, poultry, fish, legumes, non-tropical vegetable oils, and nuts, while limiting sweets and sugar-sweetened beverages, and red meats; 2. Adopt the DASH nutritional plan or Mediterranean nutritional plan if hypertension or additional cardiometabolic risk factors are present; 3. Limit saturated fat to 5–6% of total caloric intake; and 4. Limit daily sodium intake to ≤ 2400 mg for individuals with hypertension.	Encourage at least 150 min per week of moderate-intensity physical exercise beginning as soon as possible following acute spinal cord injury. The 150-min-per-week guideline can be satisfied by sessions of 30–60 min performed three to 5 days per week, or by exercising for at least three, 10-min sessions per day.
Overweight or obese	Reduce body fat mass to achieve a BMI ≤ 22 kg/m^2^		
Insulin resistance, pre-diabetes, or diabetes	Reduce FBG to ≤ 100 mg/dL and HbA1c < 7%		
Dyslipidemia	Reduce TG to ≤ 150 mg/dL and increase HDL-C to ≥ 40 mg/ dL (male) and ≥ 50 mg/dL (female)		
Hypertension	Reduce BP_SYSTOLIC_ to < 130 mmHg and BP_DIASTOLIC_ to < 85 mmHg		

### Physical Activity and Exercise in the Lifestyle Plan

Chronic spinal cord injury (SCI) increases morbidity and mortality associated with cardiovascular [77] and metabolic diseases [78], and in persons without SCI the established risk factors for these conditions are effectively managed by engaging in regular physical activity [79–81]. However, the evidence is less clear for persons with SCI, who have a range of additional physiological perturbations and barriers to physical activity, which ultimately influence adaptive responses. These issues are, in part, summarized in the Disability-Associated Low Energy Expenditure Deconditioning Syndrome (DALEEDS) model [82], including a range of disability-associated personal and environmental barriers as antecedents to deconditioning, but also accelerated physiological deconditioning in response to physical inactivity. Of particular note, persons with SCI experience a loss of innervation to skeletal muscle, resulting in a rapid and dramatic loss of previously healthy muscle mass below the level of the lesion [83], particularly among larger muscles of the lower limb.

These post-injury adaptations lead to substantial reductions in total energy expenditure, characterized by reductions in both resting metabolic rate [84] and, importantly, a reduction in physical activity energy expenditure [85]. Indeed, persons with SCI appear to perform little or no physical activity [86–89], which is likely a cause of the higher prevalence of cardiometabolic disease in this population [90, 91]. Cross-sectional studies conducted ~ 20 years ago [25, 92] placed persons with chronic SCI near the lowest end of the human physical activity and fitness spectrum. These findings were recently reaffirmed using validated objective measures of physical activity energy expenditure [85] and physical fitness [93].

Among other health organizations, the WHO has produced general physical activity guidelines for humans, recommending at least 150 min/week of moderate-intensity aerobic activity (or 75 min/week of vigorous-intensity aerobic activity), plus muscle-strengthening activities twice per week [94]. However, WHO recognize that these guidelines were not specifically tailored to the SCI population, stating that: “*These recommendations can be applied to adults with disabilities. However, they may need to be adjusted based on individual exercise capacities and specific health risks or limitations*.” Disappointingly, studies of people with SCI were excluded from the systematic reviews underpinning these public health physical activity guidelines (e.g. [95],). Consequently, the potential risks of SCI-specific adverse events, including upper-limb over-use injuries [96], skin breakdown [97], autonomic dysreflexia [98], and hyperthermia [99], were not considered in the design of the exercise guideline. Furthermore, these guidelines did not account for the perceived psychosocial and environmental barriers to engaging in physical activity, particularly the access-related barriers, which are unique to persons with disabilities [43, 69]. Coupled with the traditional hindrances of time, knowledge, and motivation, this further complicates both prescription implementation and robust exercise compliance [46, 100]. Given the specific risks and barriers to exercise among persons with SCI and the fact that more than two million people currently live with SCI worldwide, it is a public health priority to develop evidence-based physical activity guidelines for the prevention of cardiometabolic diseases in this population.

One of the significant challenges to this ambition has been the lack of high quality randomized controlled trials to provide a robust empirical evidence base. Notably, the first significant attempt to conduct a systematic review of the available evidence [101] concluded that: “*Evidence is insufficient to determine whether exercise improves carbohydrate and lipid metabolism disorders among adults with SCI.*” Several years later, despite significant remaining reservations about the quality of the evidence, Martin Ginis and colleagues [102] were able to generate physical activity guidelines. The recommendation was that adults with SCI should engage in: (i) at least 20 min of moderate-vigorous intensity aerobic activity, twice per week and (ii) strength training exercise (3 × 8–10 reps) of each major muscle group, twice per week. However, these guidelines were predominantly aimed at enhancing physical fitness, noting that: “*… although the link between fitness and improved health (through risk factor modification) may appear intuitive, the consensus panel felt that there was insufficient evidence to justify PA recommendations related to reducing disease risk.*” Given the substantial contributions to the relevant literature since this time, we provide an overview of the current evidence and the latest consensus on physical activity and exercise guidelines.

Unfortunately, the selection of practical exercise activities for persons with SCI is somewhat limited (i.e., upper-extremity exercise), and the consequences of imprudent exercise can be more severe than those experienced by persons without a disability. It is therefore essential to identify exercise activities that reduce risks of physical dysfunction and all-cause cardiometabolic disease while not increasing injury risks or hastening musculoskeletal deterioration. There is clear evidence that upper-extremity moderate-intensity continuous training (MICT) exercise improves cardiorespiratory fitness, and that the magnitude of increase depends on the level of spinal lesion and training stimulus [103–105]. However, the role of exercise in reducing cardiometabolic component risk factors in persons with SCI is less clear. In an attempt to assess the efficacy of the 2011 physical activity guidelines for improving cardio-endocrine risks in persons with SCI [102], Totosy de Zepetnik and colleagues [106] conducted a randomized controlled trial. During this 16-week training study, the intervention group completed ≥ 20 min of moderate-vigorous aerobic exercise (rating of perceived exertion 3–6 on a 10-point scale) and 3 × 10 repetitions of upper-body strengthening exercises (50–70% one repetition maximum) two times per week. Despite good adherence, following the physical activity guidelines was insufficient to improve many markers of CMS risk. These findings are in contrast to some other studies, which demonstrate that as little as 20 min of moderate-intensity exercise, performed three times weekly, in persons with SCI, improves plasma high-density lipoprotein (HDL) concentrations [104]. Further studies have also reported ~ 10% increases in HDL and a 26% decrease in low-density lipoprotein (LDL) concentration, with trends for non-significant decreases in plasma total cholesterol and TG concentrations following 3 months of vigorous intensity armcrank ergometry when conducted three times per week for 45 min at ~ 75% HR_max_ [107, 108]. Interestingly, a more recent randomized clinical trial (RCT) revealed significant and clinically meaningful effects on fasting insulin sensitivity when persons with paraplegia performed 4 × 45-min moderate-intensity (60–65% peak oxygen uptake (VO_2_peak)) arm-crank exercise sessions per week for 6 weeks [105]. This study concluded that, while the intervention was able to enhance indices of hepatic insulin sensitivity, there was no effect on markers of peripheral insulin sensitivity. It seems clear from these studies that, in order to observe significant effects on cardiometabolic component risks, the absolute volume (135–180 min per week) and intensity (60–70% VO_2_peak) of MICT has to be substantially higher than previously recommended. This finding is probably not surprising given the relatively small muscle mass involved in upper body exercise and the somewhat limited potential to stimulate disturbances in whole-body hemodynamic or metabolic homeostasis.

As a consequence of this evidence and observations in non-injured humans, there has been considerable interest in the efficacy of alternative forms of higher intensity upper-body exercise (i.e., high-intensity interval training, HIIT) for persons with SCI [109]. The primary rationale for HIIT is that it allows a higher volume of vigorous-intensity exercise to be accrued in a single exercise session. When compared to light- and moderate-intensity continuous exercise training, vigorous-intensity physical activity is more effective in reducing the risk of cardiovascular [110, 111] and all-cause mortality [112–114] in non-injured humans. There is also mounting evidence from studies in non-SCI cohorts that HIIT promotes superior peripheral [115] and whole-body physiological adaptations [116, 117], which would be of specific value in overcoming the numerous training limitations for persons with SCI. While a wide range of HIIT protocols have been described in the literature, the terminology proposed by Weston et al. [118], is particularly helpful, where HIIT protocols adopt exercise intensities between 80 and 100% of V̇O_2_ peak and those protocols using “all-out” efforts, or efforts > 100% V̇O_2_ peak are referred to as “sprint interval training” (SIT). It is relatively simple to deliver such a training stimulus via upper-limb armcrank exercise for persons with SCI. Indeed, early indications are that persons with SCI experienced greater enjoyment with HIIT and SIT protocols compared with MICT [119]. Further robust studies into the efficacy of HIIT for reducing cardiometabolic component risks in the fasted and post-prandial states are underway (e.g. [120],) and are necessary to confirm the benefits of HIIT in persons with SCI.

**Fig. 2:**
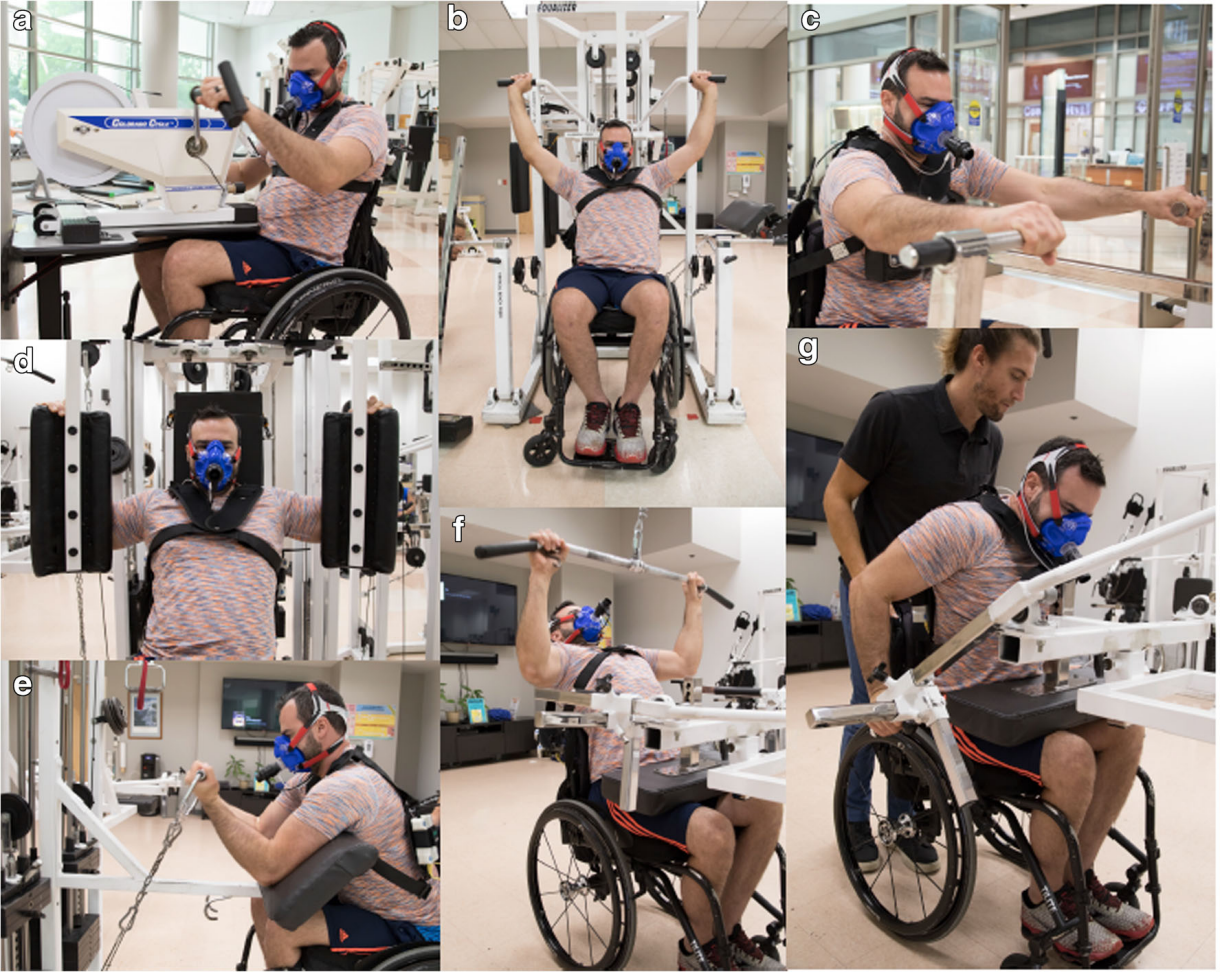
Images of a participant completing the various elements of the circuit resistance training (CRT) protocol: **a** arm crank ergometry, **b** military press, **c** horizontal row, **d** pectoralis (“pec”) dec, **e** preacher curl, **f** wide grip latissimus pull-down, and **g** seated dip

Resistance exercise training is now also universally recommended in exercise guidelines, adopted for use by persons with a disability [65]. Resistance training offers the potential to both prevent and treat shoulder pain [121] while improving or maintaining transfer and propulsion independence. One of the earliest studies to assess the efficacy of upper-body resistance training in men with incomplete low thoracic spinal lesions had a particular emphasis on developing triceps strength (for elbow extension during crutch walking) was undertaken for 7 weeks. In addition to the expected gains in triceps brachii strength, significant increases in V̇O_2_max were also observed following training [122]. These findings have since been confirmed in more recent studies in persons with SCI [123, 124], stimulating interest in the efficacy of resistance training and mixed-modality training protocols for enhancing cardiometabolic biomarkers. Indeed, strength and aerobic improvements can both be obtained using a “circuit resistance training” (CRT, Fig. 2) approach to integrating cardiorespiratory and resistance training exercise [125, 126]. Interestingly, this same circuit protocol was later shown to be effective at improving the atherogenic lipid profile of persons with paraplegia [127]. More recently, this circuit resistance protocol has been adapted for use by persons with tetraplegia, for whom both increased strength and endurance were reported when 6 months of training was accompanied by immediate post-exercise whey protein supplementation [128], a technique used to enhance glycogen replenishment following exercise carbohydrate and amino acid depletion [129]. The circuit resistance training (CRT) protocol has also been made compatible for home and community integration by use of elastic bands [130] and has been recommended by the American Physical Therapy Association as part of their Physical Fitness for Special Populations Program for Individuals with SCI.

To summarize, comprehensive physical activity guidelines to enhance cardiometabolic component risks for persons with SCI were recently updated and published by several authorities [65, 131, 132]. Reassuringly, these latest recommendations have considerable commonality, promoting both cardiorespiratory exercise and resistance exercise training, as well as highlighting the importance of avoiding inactivity. Quite rightly, the Consortium for Spinal Cord Medicine Clinical Practice Guideline [65] emphasizes the importance of higher volumes (150 min per week) and higher frequencies (up to 5 days per week) of exercise for delaying the progression of cardiometabolic disorders. Further research is beginning to demonstrate the benefits for specific forms of higher intensity armcrank ergometry and mixed-mode resistance exercise, and it is likely that these activities will inform the development of future iterations of exercise guidelines. Given the limited impact of upper-body MICT on physiological responses and physical activity energy expenditure, conditioned adults with SCI should be encouraged to accrue their weekly exercise dose by engaging in higher intensity forms of intermittent upper-body exercise (e.g., higher intensity interval training (HIIT)), including continuous resistance training (CRT).

## Other Interventions for CMS Risks

### Secondary Management: Pharmacotherapy

While comprehensive lifestyle intervention is the primary approach for CMS control, a failure to satisfy targets using the combination of exercise conditioning and nutritional control then defaults to pharmacotherapy as secondary management (Table 5). These approaches address individual risk components of the CMS, and in most instances, selection of a therapeutic agent for the PVA Guideline was made by guideline approaches and good medical practices adopted for the non-disabled population (Table 5). For example, hypertension pharmacotherapy in the PVA Guidelines was based upon the Eighth Joint National Committee (JNC 8) evidence-based guideline for the management of high blood pressure [134]. Control for dysglycemia was consistent with the ADA standards of medical care for type 2 diabetes [133]. The sole area where medicines were not recommended was for treatment of obesity, where available agents have not been systematically tested for safety and tolerance in the SCI population, risks may outweigh potential benefits, and drug interactions with other prescription and non-prescription medicines may be hazardous. The latter was specifically cited for the potential risk of serotonin syndrome

**Table 5:** Risk targets and first-line recommendations for management of CMS using pharmacotherapy

Risk	Goal	Secondary management: pharmacotherapy
CMS diagnosis	As above	Treat specific CMS risk component
Overweight or obese		None recommended
Insulin resistance, pre-diabetes, or diabetes		Metformin (glucophage) as the first-line agent for treatment of HbA1c > 7%, unless contraindicated or poorly tolerated. If the maximum tolerated dose of Metformin fails to achieve goals, add a second and possibly a third agent, according to ADA Standards of Medical Care [133].
Dyslipidemia		Guide patient selection for pharmacotherapy by other factors commonly seen in SCI, such as low levels of HDL-C and high levels of C-reactive protein. Initiate statin monotherapy using at least a moderate-intensity statin (e.g., rosuvastatin 10 mg/day).
Hypertension		JNC 8 guidelines [134] recommend initial antihypertensive treatment with a thiazide-type diuretic, calcium channel blocker (CCB), angiotensin-converting enzyme inhibitor (ACEI), or angiotensin receptor blocker (ARB) in the non-Black population, and either a thiazide-type diuretic or CCB in the Black population.

.

### Bariatric Surgery

Bariatric surgery has become a routine, yet still aggressive approach for clinical management of morbid obesity. However, limited study has systematically tested the safety and effectiveness of bariatric surgery in persons with SCI, and while several case reports have described the procedures [135–137], inadequate information has documented perioperative or post-operative risks that are unique to the population. Further, guidelines for determining bariatric surgery candidacy in non-disabled individuals have limited relevance for the SCI population [138, 139] and do not address the complex needs/risks including post-operative mobility and activities of daily living deficits. Otherwise, risks of neurogenic bradycardia, neurogenic hypotension, adapted myocardial atrophy, circulatory hypokinesis, autonomic dysreflexia, neurogenic restrictive and obstructive lung disease, neurogenic bladder and bowel, neurogenic skin, sarcopenia, osteopenia/osteoporosis, and spasticity are noted in the PVA Guideline [65].

## Conclusions

An alarming number of individuals with SCI develop component risks for CMS at some point within their lifespan, the two most serious of which are sarcopenic obesity and insulin resistance. For many individuals with SCI, exercise offers an effective strategy for attenuation of these risks, with a benefit favored by the adoption of more intensive activity. The value of this exercise in CMS/CVD management may be less useful for individuals with higher levels of injury where functional sympathectomy has been sustained. In these individuals, when combined with balanced, calorie-regulated nutrition, the two modifications constitute a lifestyle intervention that favors a best-practice appropriate for disease management. When lifestyle intervention is ineffective for risk reduction, both pharmacotherapy and bariatric surgery become options for CMS risk abatement, but may also be accompanied by unique risks and variable benefits for the SCI population.
